# Immunology of Plasmacytoid Dendritic Cells in Solid Tumors: A Brief Review

**DOI:** 10.3390/cancers11040470

**Published:** 2019-04-03

**Authors:** Vladimír Koucký, Jan Bouček, Anna Fialová

**Affiliations:** 1 Sotio, 17000 Prague, Czech Republic; fialova@sotio.com; 2 Department of Otorhinolaryngology and Head and Neck Surgery, First Faculty of Medicine, Charles University and Motol University Hospital, 15006 Prague, Czech Republic; jan.boucek@fnmotol.cz

**Keywords:** plasmacytoid dendritic cells, cancer, tumor microenvironment, tumor immunology

## Abstract

The immune response, both innate and adaptive, is a key player in cancer development and progression. Plasmacytoid dendritic cells (pDCs) are a subset of dendritic cells that play one of the central roles in the immune system. They are known mostly as the major IFN type I-producing cells upon stimulation of Toll-like receptors 7 and 9. However, based on current knowledge, the functionality of pDCs is very complex, as they have the ability to affect many other cell types. In the context of the tumor tissue, pDCs were mostly described to show substantial functional defects and therefore contribute to the establishement of immunosuppressive tumor microenvironment. Immunotherapeutic approaches have proven to be one of the most promising treatment strategies in the last decade. In view of this fact, it is crucial to map the complexity of the tumor microenvironment in detail, including less numerous cell types. This review focuses on pDCs in relation to solid tumors. We provide a summary of current data on the role of pDCs in different tumor types and suggest their possible clinical applications.

## 1. Introduction

The tumor microenvironment is a complicated system of cells that creates an extensive network of interactions. Immune cells form a crucial part of this network and have a huge impact on the development and progression of the disease. Most of the studies concerning cancer immunology have focused on T cell-mediated immune responses, and the positive prognostic value of tumor-infiltrating CD8^+^ T cells has been established in many oncologic diseases. In addition, the success of immune checkpoint inhibitors, namely, anti-CTLA-4 and anti-PD-1 monoclonal antibodies, in clinical trials has produced a new dose of optimism for immunooncology. However, in most malignancies, a significant proportion of patients remain unresponsive to T cell-targeted approaches, and further research on the tumor microenvironment is needed. 

In addition to T cells, dendritic cells (DCs) have long been one of the main targets in cancer research. Generally, DCs are one of the central components of the immune system responsible for the initiation of adaptive immune responses. Plasmacytoid dendritic cells (pDCs), a less explored subgroup of DCs, are mostly examined in relation to viral infections and autoimmune diseases, and have been rather disregarded in immunooncology thus far. Primarily, pDCs are known as the most potent producers of interferon α (IFNα) from all of the peripheral blood cells [1]. The main stimuli for secretion were found to be viral nucleic acids or self-derived DNA [2]. Nucleic acids interact with the most important pDC sensing receptors, namely, Toll-like receptors 7 and 9 (TLR7, TRL9), resting in endosomes. After TLRs are triggered, the pDC-derived IFN type I production is dependent on a complex containing MyD88 and IRF7. Within the complex IRAK1 and/or IKKα phosphorylates IRF7, which is translocated into the nucleus and so can regulate the expression of IFNα and other type I IFNs, namely, IFNβ, IFNτ and IFNω [3]. Moreover, the constitutive high expression of IRF7 is a particularity of pDCs that allows them to secrete very rapidly significant amounts of IFNα and β [4] However, type I IFNs are neither the only products of pDCs nor their only possible mechanism of interaction with other players in the tumor microenvironment. Indeed, the MyD88-IRAK4-TRAF6 complex drives NF-κB-dependent inflammatory cytokine induction. The secretion of IL-6, IL-10, TNFα and IFNγ has been reported [5,6]. In addition, the production of chemokines CCL3, CCL4, CCL5, CXCL9, and CXCL10 has been observed as these substances play a role in migration of pDCs themselves and also attract other innate immune cells, such as NK cells and macrophages [7,8]. Furthermore, other functions have been assigned to pDCs, such as antigen presenting function, T regulatory lymphocytes (Tregs) induction, cell-to-cell contact-dependent cytotoxicity, and interactions with NK cells and B cells [5,9,10,11]. Therefore, in this article, we aimed to summarize the current knowledge of the role of pDCs in the immunology of solid tumors.

## 2. Overview of pDC Biology 

Plasmacytoid DCs count for less than 1% of peripheral blood mononuclear cells (PBMCs) in healthy individuals. They can be generated from both common myeloid and common lymphoid progenitors, including substream pathways with various potential to give a rise to pDCs [12]. Plasmacytoid DCs are fully developed in bone marrow, released into peripheral blood as immature (non-activated) cells, and then enter secondary lymphoid organs and peripheral tissues, including pathologic conditions such as tumors, inflammatory and autoinflammatory lesions. In general, the major growth factor for DC development is the fms-like tyrosine kinase 3 ligand (FLT3-L) [13,14]. The FLT3 receptor is expressed on pDC progenitors, and as shown in mouse models with FLT3 deletion, the pDC population is far more dependent on this pathway than the conventional DC (cDC) population [15]. The main transcription factor, both in mice and humans, for differentiating progenitors into the pDC branch and in maintaining the pDC phenotype, is E2-2 [16]. E2-2 is activated through the FLT3 receptor via the STAT 3-dependent mechanism [17]. The interruption of E2-2 expression in mature pDCs leads into their spontaneous differentiation into cDC-like cells [18]. 

Unlike cDCs, pDCs enter T lymphocyte-rich areas of lymphatic organs directly from the blood through high endothelial vessels (HEVs) [19]. The main receptors driving migration into the secondary lymphatic organs are L-selectin CD62L and CCR7. The latter is upregulated upon TLR9 stimulation and interacts with chemokines CCL19 and CCL21 produced by fibroblastic reticular cells, the stromal cells in T cell zones of lymph nodes [19,20]. Additional important pathways that lead pDC migration in homeostatic and pathologic conditions are CXCR4/CXCL12 and CXCR3/CXCL9/CXCL10/CXCL11 [21,22]. CXCL12 and ligands of CXCR3 are expressed in both secondary lymphoid organs and in epithelium inflamed either due to a viral infection or due to a malignant transformation [21,23]. It has been reported that the capability of the chemokine CXCL12 to enhance the recruitment of pDCs was conducted by CXCR3 ligands, induced under inflammatory conditions [23]. At least partial dependency on the CXCR4/CXCL12 pathway was demonstrated in melanoma, ovarian cancer, and head and neck squamous cell carcinoma [21,24,25]. This result implies that other receptors, including CCR6 or CCR7, might play a role in pDC migration to the tumor sites [26,27].

Originally, pDCs were named “plasmacytoid monocytes” and characterized as CD4^+^ CD123^+^ cells that were negative for lineage markers of B cells, T cells, NK cells, and monocytes [28]. Since then, specific surface markers that facilitate the analysis and isolation of these cells from blood and peripheral tissues have been described. A C-type lectin blood DC antigen 2 (BDCA-2) is a commonly used marker for pDC identification. Nevertheless, there are drawbacks in its use for pDC enrichment, because engagement of the receptor with an antibody leads to a decrease of pDC functional capacity [29]. Therefore, a blood dendritic cell antigen 4 (BDCA–4) is used for pDC enrichment [30]. BDCA-4 is identical to neuropilin-1 (NP-1), a receptor known to be expressed on other non-hematopoietic cells, such as neurons and some tumor cells. The standard markers used to identify pDC in mice are B220, Ly6C, BST2, Siglec-H and CD11c [31].

Human plasmacytoid DCs can be further divided into minor subpopulations with variability in functional characteristics. The CD2^high^ pDC subpopulation showed expression of lysozyme, secreted more IL12p40, and was more efficient in triggering proliferation of naïve T cells [32]. Moreover, Zhang et al. further diversified CD2^high^ subsets and identified CD2^+^CD5^+^CD81^+^ pDCs in blood, bone marrow, and tonsils [33]. This subset produced little IFNα upon TRL stimulation; however, in addition to T cell proliferation, this subset also promoted plasma cell differentiation. Recently, Alculumbre et al. reported three subsets based on PD-L1 and CD80 expression. Cells with plasmacytoid morphology, specialized in type I IFN production expressed PD-L1 but not CD80. Cells negative for PD-L1 but positive for CD80 showed dendritic morphology and adaptive immune functions. As expected, double positive PD-L1^+^CD80^+^ pDCs showed both innate and adaptive functions [34]. Other pDC subpopulations were defined based on CXCL10 expression and/or IFNα secretion, however their specific functions are yet to be explored [35,36].

The most important secretory products of pDCs are type I IFN, especially IFNα. Type I IFN are cytokines with a pleiotropic effect reported to have a bipolar role in anticancer immunity [37]. These cytokines can increase NK cell and CD8^+^ T cell cytotoxicity, stimulate maturation of DCs and promote proinflammatory activation of macrophages [37,38]. Consequently, IFNα2A and IFNα2B were approved for the treatment of several malignancies [39]. However, prolonged exposure to type I IFN can provoke immunosuppression through enhanced IL-10 production by Tregs and induction of indoleamine-2,3-dioxygenase (IDO) in DCs [37]. It is not surprising that because of the systemic importance of type I IFN, their production is under multiple surveillance. The important “checkpoint” molecules expressed on human pDCs that were reported to negatively regulate IFN secretion are NKp44, TIM-3, BDCA-2, ITL7, CLEC4A and LAIR1 [29,31,40,41,42,43].

## 3. Negative Role of pDCs in Solid Tumors

The natural behavior of tumors is to escape from the immune system reaction by many mechanisms that can be modified or developed through the course of the disease. Tumors can lose their antigenic or immunogenic potential and induce an immunosuppressive environment. The latter is orchestrated by the production of immunosuppressive cytokines (IL-10, TGFβ, and PGE2), recruitment of Tregs, activation of negative regulatory pathways (CD80/CTLA4, PD-1/PD-L1), or overexpression of immunomodulatory enzymes (IDO) [44]. In both humans and mice, tumor-infiltrating pDCs were shown to be mostly defective in their functions and were supposed to support the immunosuppressive setting of the tumor microenvironment [45]. Moreover, pDCs were identified as a negative prognostic markerin breast, ovarian, and oral cancers [46,47,48]. However, given that pDCs are a rather sparse population in the tumor tissue, only a limited number of studies have analyzed the function of pDCs isolated directly from human tumors. 

### 3.1. Dysregulation of pDC Functions in the Tumor Microenvironment

A decreased capacity to produce IFNα is considered as the main indicator of pDC dysfunction. Hartman et al. reported a diminished capacity of pDCs derived from head and neck squamous cell carcinoma (HNSCC) to produce IFNα upon CpG stimulation compared to blood-derived pDCs [49]. A possible mechanism of this phenomenon was suggested to be a decrease in TLR9 expression [49]. The same author group later elucidated the underlying mechanism of impaired IFNα secretion, identifying prostaglandin E2 and TGFβ from HNSCC culture supernatants as the most important negative regulators of pDC functions [50]. Other research teams reported similar effects on IFNα secretion in the setting of HNSCC when blood-derived human pDCs were incubated with tumor supernatants [51,52]. Bruchhage et al. also analyzed cytokines in HNSCC supernatants and tested their effect on pDCs. In this study, IL-10 was identified as one of the major actors that impaired IFNα secretion [51]. Similarly, tumor-associated pDCs showed decreased IFNα secretion upon TLR7 and TLR9 stimulation in breast and ovarian cancers [52,53]. However, in comparison to HNSCC, different mechanisms explaining this phenomenon were observed, as the most important soluble factors in the tumor microenvironment responsible for the impaired function were designated TNFα and TGFβ, but not IL-10 [54]. Indeed, TGFβ and TNFα in a synergistic manner negatively affected IRF-7 expression and thus inhibited the IFNα secretion pathway. Moreover, to support the significance of this IFN-regulating pathway in breast cancer, higher expression of IRF-7-regulated genes in primary tumors of breast cancer patients positively correlated with prolonged bone metastasis-free survival [55]. The important role of TGFβ was also demonstrated by Terra et al. In TC1 and B16-OVA mouse models, TGFβ was identified as the main cytokine suppressing IFNα production by tumor-associated pDCs [56].

Additionally, immunoglobulin-like transcript 7 (ILT-7) signaling was shown to play a role in the negative regulation of type I IFN production by pDCs [57]. However, the phenomenon was demonstrated by the interaction of CpG-stimulated blood-derived pDCs with ILT-7 ligands-expressing human cancer cell lines and not in patient tumor tissue. In human melanoma invaded lymph nodes and skin metastases, higher infiltration of the LAG3^+^ pDC subpopulation compared to blood was reported. Moreover, LAG3-mediated activation of pDCs led to low production of IFNα but high secretion of IL-6, suggesting an important role for this alternative activation pathway in establishing the immunosuppressive microenvironment [58]. Functionally impaired pDCs were also identified in preneoplastic lesions of the uterine cervix [59]. Based on in vitro experiments, Demoulin et al. showed low IFNα secretion by pDCs upon cocultivation with cervical cancer cell lines infected by human papillomavirus 16 (HPV16) [60]. In this study, high mobility group B1 protein (HMGB-1) produced by neoplastic keratinocytes was identified as an important negative regulator of IFNα secretion. In contrast, HIV1-stimulated blood-derived pDCs from healthy donors secreted HMGB-1, which increased IFNα production in an autologous loop [61]. In view of the fact that pre/malignant lesions of the cervix are associated with chronic infection with high-risk HPV, it is worth noting that blood circulating pDCs were also shown to react by IFNα secretion upon stimulation with HPV16 capsid-derived virus-like particles, although the amount of IFNα was lower than upon CpG 2216 stimulation [59]. This demonstrates a possible “fight” between the immunomodulatory capacity of the tumor cells and the stimulatory effect of viral antigens on pDCs. Therefore, the pDC phenotype and function might differ in virus-associated tumors and tumors of other etiologies.

### 3.2. Pro-Tumorigenic Effects of pDC 

The many times reported mechanism by which pDCs augment the immunosuppressive environment is the induction of Tregs through the ICOS/ICOS-L pathway or indoleamine 2,3-dioxygenase (IDO) expression [45]. Interestingly, unlike tolerogenic DCs, immunosuppresive pDCs do not have to show an immature phenotype, but can even be stimulated by TLR9 agonists. Tumor-infiltrating Tregs produce IL-10 and TGFβ, which further support tumor progression. As described above, IL-10 and TGFβ could impair the capacity of pDCs to produce IFNα and potentially create a vicious cycle intensifying the immunosuppressive effect of the tumor. The importance of ICOS-L^+^ pDCs in the induction of Tregs was reported in human melanoma, breast cancer, ovarian cancer, and liver tumors [53,62,63,64]. Furthermore, in a human ovarian cancer study, high densities of pDCs and ICOS^+^ Foxp3^+^ Tregs were found to be strong predictors of disease progression [62]. A positive correlation between the proportions of tumor-infiltrating pDCs and Tregs was also found in glioma, thyroid gland cancer, and gastric cancer [65,66,67]. In contrast, the only study which is in a discrepancy with these reports and concerns tumor-infiltrating cells is focused on colorectal cancer (CRC) [68]. In CRC high densities of Tregs were shown to be rather a positive prognostic marker [69,70]. Thus, Tregs in CRC are supposed to have anti-tumorigenic effect helping to suppress undesirable chronic inflammation in the tumor tissue. Positive correlation of functionally impaired pDC and Tregs may therefore occur in tumors, where a suppressive microenvironment prevails and Tregs support these protumoral conditions. 

In addition to ICOS-L stimulation, costimulation by OX40L expressed on pDCs led to the polarization of the immune response towards the Th2 direction in a melanoma mice model [64]. Consistent with these results, increased levels of OX40L^+^ pDC and Th2 T cells were detected in the peripheral blood of patients with advanced stages of melanoma [64]. In a mouse model of melanoma, IDO^+^ pDCs derived from tumor-draining lymph nodes were reported to stimulate CD4^+^CD25^+^Foxp3^+^ Tregs. These Tregs subsequently caused upregulation of PD-L1 and PD-L2 on DCs and promoted the immunosuppressive microenvironment [71]. This effect was abrogated when IDO-KO mice were used. This study suggests that the effect of PD-1 and PD-L1 inhibitors may be augmented by targeting the negative action of tumor infiltrating pDCs. To support this hypothesis, Ray et al. showed in multiple myeloma (MM) patients that blockade of the PD-1/PD-L1 axis in pDCs and in cocultures with CD8^+^ and CD4^+^ T cells caused an increased proliferation rate of T lymphocytes [72]. Moreover, using an anti-PD-L1 antibody in a pDC coculture with autologous NK cells from MM patients restored NK cell cytolytic activity against GFP^+^ MM.1S cells.

In addition to induction of Tregs, there are sporadic reports about other tumor-promoting functions of pDCs. Curiel et al. described the induction of neoangiogenesis via TNFα and IL-8 production by CD40L-activated pDCs derived from human ovarian tumor ascites [73]. Another partially proangiogenic and proinvasive cytokine, IL-1α, was reported to be produced by pDCs from human non-small cell lung cancer tissue [74]. However, IL-1α is an ambivalent cytokine with both pro- and antitumoral effects. In addition to the above-mentioned mechanisms by which pDCs contribute to the induction and maintenance of the immunosuppressive tumor microenvironment, granzyme B secreted by IL-3-stimulated pDCs was reported to be able to decrease both CD4^+^ and CD8^+^ T cell proliferation [75]. This phenomenon was further enhanced by IL-10 but inhibited by TLR stimulation. Because of the aforementioned negative role of pDCs in many tumors, the possibility of pDC depletion could be a way to release a local immune response. Sawant et al. reported a decrease in tumor burden and metastatic spread after pDC depletion in a breast cancer mouse model using an anti-PDCA-1 antibody [76]. In a glioma mouse model, pDC depletion led to prolonged survival, decreased tumor-infiltrating Treg numbers, and substantially decreased production of IL-10 in the remaining intratumoral Tregs [65].

**Figure 1:**
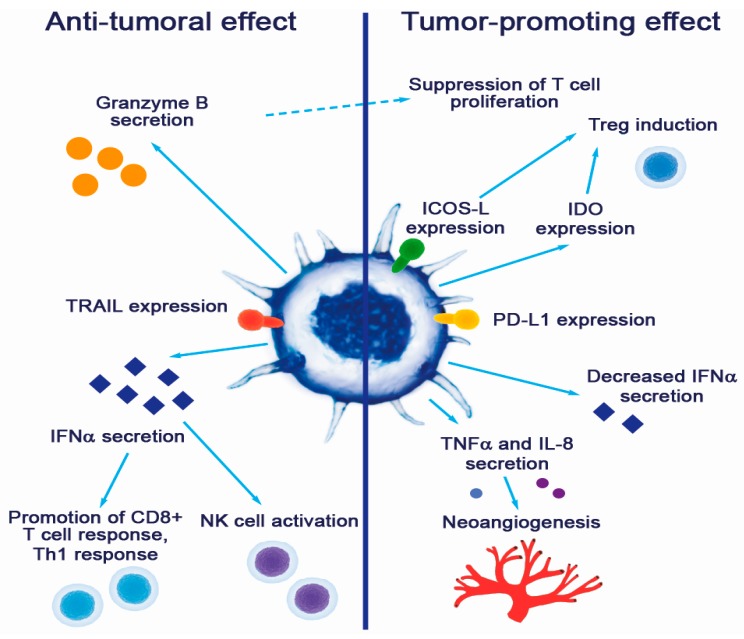
Contribution of pDCs to cancer pathogenesis.

Plasmacytoid DC contributions to tumor promotion and suppression are summarized in Figure 1.

Most of the studies concerning the role of pDCs in the tumor microenvironment lack the functional analysis of pDCs or the significance in correlation with clinical parameters of the patients. Studies that showed a statistically significant prognostic impact of pDCs in cancer patients are listed in Table 1. Reported pathogenetic roles of pDCs in different tumor types are listed in Table 2.

## 4. Anti-Tumorigenic Capacity of pDCs in Tumors and Possible Use in Cancer Therapy 

In contrast to the negative impact of pDCs in tumors, there are only sporadic reports concerning the positive prognostic role of pDCs in cancer patients. Bailur et al. reported that high levels of pDCs in breast cancer patients correlated with prolonged overall survival [77]. Moreover, the patient’s group with a low MDSC/pDC ratio and a CD8^+^ T-cell response to HER2 peptide stimulation in vitro had a 100% 5-year survival rate. Compared to the aforementioned study of Treillux et al. reporting negative prognostic role of pDCs, this study explored circulating pDCs. Nevertheless, Wu et al. observed a significant tumoricidal activity of pDCs in the TUBO breast cancer cell line [82]. The CpG- or IMQ-stimulated pDCs were able to lyse tumor cells in vitro and reduced tumor burden in an experimental mouse model. The effect was assigned to increased TRAIL and granzyme B expression and was stronger upon IMQ stimulation. However, the cytotoxic activity of pDCs was reduced but did not vanish completely after the blockade of these molecules, leaving space for other unexplored mechanisms. The importance of pDC-derived IFN type I produced upon TLR7 stimulation was reported by Le Mercier et al. [83]. In orthotopic breast tumor mouse model using NEU15 cell line, the IFN type I was the essential factor responsible for tumor regression in vivo. Interestingly, the authors did not observe any increase in TRAIL expression upon TLR7 stimulation. Encouraging data concerning pDC cytotoxic activity were also observed in melanoma. Melanoma is an immunogenic tumor that is well known for a high immune infiltration and therefore creates a valuable model for testing novel immunotherapeutic approaches. In cytotoxic assays using melanoma cell lines SKMel2 and WM793, pDCs activated by IMQ or recombinant IFNα were able to lyse tumor cells [84]. These findings were confirmed by other teams in mouse models, highlighting the essential role of TLR7 and IFNαR signaling in pDC-mediated cytotoxicity [85,86,87]. Consistently, TRAIL and granzyme B were identified as the main cytotoxic agents. However, in view of the fact that pDCs do not produce perforin, the exact background of the perforin-independent granzyme B-mediated cytotoxic effect has not been satisfactorily clarified so far. Indirect antitumoral effects of pDC-derived IFN type I were also reported in B16 melanoma mouse model [88]. Liu et al. showed that CpG-activated pDCs could recruit NK cells and enhance their cytotoxic activity. Moreover, IFNγ and perforin produced by activated NK cells were described to be important factors in CD8^+^ T cell cross-priming. Taken together with the fact that pDC are potent enhancers of the cDC capacity to prime antigen-specific CD8+ T cells responses [89], these findings suggest possible linking of innate and adaptive immunity by activated pDCs 

Although pDCs constitute a minor population of immune cells in the circulation and in the tumor microenvironment, based on the complexity of their interactions, pDCs might be a promising target for cancer immunotherapy. 

Administration of recombinant IFNα is an approved immunotherapeutic approach in renal cell carcinoma, melanoma, and AIDS-related Kaposi sarcoma. However, the overall response rate is quite low and undermined by serious toxic side effects, especially hematologic toxicity, flu-like syndrome, or anorexia, even in the more tolerable PEGylated form [39]. Interferon α was a standard monotherapy or combinational therapy with bevacizumab in advanced and metastatic renal cell carcinoma in patients with a good performance status who underwent cytoreductive nephrectomy [90]. In melanoma the use of IFNα is applicable as an adjuvant treatment for patients with resectable AJCC stage III disease [91]. Although new treatments, such as tyrosine kinase inhibitors and immune checkpoint inhibitors, are replacing IFNα, there are attempts to combine IFNα with current immunotherapy. The combination has a biological rationale, as IFNα is known to induce the expression of PD-L1, potentially leading to greater inhibition of tumor growth and increased tumor cell apoptosis [92]. In the phase Ib KEYNOTE-029 study a combination of IFNα with pembrolizumab was evaluated in melanoma and renal cell carcinoma patients [93]. However, serious grade toxicity was reported in 59% of patients, and the maximal tolerated dose had only limited antitumor activity. Therefore, an emphasis should be put on the targeted delivery of the active substance directly into the tumor environment to enhance the immune response and effect of checkpoint inhibition [38]. One of the possible approaches is to stimulate producers of IFNα at the tumor site via the intralesional application of TLR9 and TLR7 ligands. CpG ODN nanorings were reported to induce the robust production of IFNα by pDCs, leading to a reduction of the tumor size in a thymoma mouse model [94]. Moreover, in a phase I/II clinical trial, intratumoral CpG injection combined with radiotherapy in 15 patients with mycosis fungoides lymphoma led to a clinically meaningful response in five subjects, finding an increase in pDC infiltration and decrease in Treg levels [95]. However, in most studies using TLR9 agonists, immunomonitoring was focused on the analysis of T cell and B cell responses but not on the contribution of pDCs. In a different approach, IFNα released by activated pDCs after adenovirus-mediated delivery of the immune-stimulatory cytokine FLT3-L led to an antitumoral effect in the glioma mouse model [96]. Adjuvant FLT3 ligand administration combined with a peptide-based vaccine and topically applied imiquimod (IMQ) was also tested in melanoma patients. Whereas FLT3 ligand treatment led to increased levels of immature myeloid DCs and pDCs in the peripheral blood, cutaneous reactions to peptide vaccination and circulating peptide-specific CD8^+^ T cells were more frequent in patients who received the peptide vaccine in combination with both the FLT3 ligand and IMQ [97]. Topical application of IMQ, a synthetic TLR7 agonist approved by the FDA for the treatment of basal cell carcinoma, is known to strongly stimulate pDCs, leading to their attraction into the skin lesions and activation of their cytotoxic activity. In melanoma mice models, IMQ alone reduced tumor lesions through production of IFNα/β, which led to TRAIL and granzyme B secretion by pDCs, opposing the possible immunosuppressive effect of granzyme B expression in pDCs [85]. In humans, a phase II clinical trial combining IMQ with monobenzone in melanoma stage III–IV patients led to the local regression of cutaneous metastases in 38% of the patients [98]. 

Active cellular therapy is another promising immunotherapeutic approach. Monocyte-derived DCs pulsed with cancer antigens already showed their potential efficacy, and there are many ongoing clinical trials. However, the antigen presenting role of pDCs in the tumor microenvironment is quite elusive. Even though pDCs are capable of antigen presentation, they are less effective than classical DCs [65]. Especially uptake of exogenous antigens by pDCs is considered to be inferior to cDCs [99]. On the other hand, when pDCs are infected by a virus, they are capable of sustained presentation of these endogenous viral antigens, a potentially favorable factor in the context of virally induced tumors. Moreover, Tel. et al. reported high capacity of human blood-derived pDCs to cross-present tumor derived antigens to CD8+ T cells in vitro, despite less antigen uptake [100]. Also, there are sporadic reports of selective pDC efficacy, as is the case for intranodal injection of pDCs loaded with tumor-associated peptides in stage IV melanoma patients. Autologous pDCs were pulsed with tumor peptides derived from the melanoma-associated antigens gp100 and tyrosinase and intranodally injected into patients [101]. Seven out of 15 patients showed a significant increase in gp100_154_-specific CD8^+^ T cell frequency, and the overall survival of the patients who received the pDC vaccine was significantly higher compared to standard chemotherapy (22 vs 7.6 months).

## 5. Conclusions

Plasmacytoid DCs affect many cell types and processes in the course of human diseases, including cancer. On the one hand, pDCs were found to infiltrate different cancer types, having mostly negative effects in the tumor microenvironment. On the other hand, in vitro and in vivo data also showed a significant antitumoral potential of adequately stimulated pDCs. Similarly to other tumor-infiltrating immune cells, the phenotype and functional capacity of pDC is dependent on the shape of the tumor microenvironment including the pattern of the immune cell infiltrate, cytokine milieu and the ability of tumor cells to successfully evade the immune response. Factors such as TNFα, TGFβ, and IL-10 abrogate the antitumoral potential of pDC and rather enhance their pro-tumorigenic effect. However, a well-designed stimulation of pDCs via TLRs may lead to their reactivation and significantly help to restore the local immune response. A precise understanding of the regulatory mechanisms that influence pDC functionality can put missing pieces in the puzzle, showing the picture of the interactions between the immune system and cancer, and thus lead to an improvement in current therapeutic approaches.

## Figures and Tables

**Table 1:** Prognostic value of pDCs in cancer patients.

Cancer Type	Prognostic Value	Functional State of pDCs	Detection Method	Reference
Breast cancer	Positive, OS	NE	FC—blood marker: CD123+	[77]
	Negative, OS, PFS	NE	IHC—tumor tissue marker: CD123+	[47]
Ovarian cancer	Negative, PFS	Induction of IL-10 producing T cells Decreased IFNα production	FC—tumor tissue marker: BDCA2+	[48]
			IHC—tumor tissue marker: CD123+	[52]
Oral cancer	Negative, OS	Decreased IFNα, IL – 6 and TNFα production	IHC—tumor tissue marker: CD123+	[46]
Melanoma	Negative, OS	NE	IHC—tumor tissue marker: CD123+	[78]
	Negative, OS	Upregulation of OX40L and ICOS-L	FC—tumor tissue marker: BDCA2+/CD123+	[64]
	Negative, OS, PFS	NE	FC—blood marker: CD123+	[79]
Pancreatic cancer	Positive, OS	NE	FC—blood marker: CD123+	[80]

OS—overall survival, PFS—progression-free survival, IHC—immunohistochemistry, FC—flow cytometry. NE—not evaluated.

**Table 2:** Pathogenetic role of pDCs in cancer.

Cancer Type	pDC Source	Pathogenetic Mechanism	Reference
Breast cancer	Human cancer tissue	Decreased IFNα production via tumor-derived TNFα, TGFβ	[54]
		Decreased IFNα production, Tregs expansion	[53]
		Increased Treg proliferation and IL-10 production	[81]
Ovarian cancer	Human blood—healthy donor	Decreased IFNα production after co-incubation with tumor-derived supernatants, suppressive role of TNFα, TGFβ	[52]
	Human cancer tissue	Immunosuppression via induction of ICOS^+^ Tregs producing IL-10, dependent on ICOS-L costimulation	[62]
	Human malignant ascites	Induction of neoangiogenesis via TNFα and IL-8 production	[73]
Cervical cancer	Human cord blood	Altered maturation and decreased IFNα production after co-incubation with cervical cancer cell lines, HMGB1 dependent mechanism	[60]
Head and neck cancer	Human cancer tissue	Decreased IFNα production upon CpG stimulation, decreased expression of TLR9	[49]
	Human blood—healthy donor	Decreased IFNα production after co-incubation with tumor-derived supernatants, suppressive role of IL-10	[51]
Melanoma	Human cancer tissue	Impaired capacity to secrete IFNα in response to TLR9, induction of Tregs via OX40L and ICOS-L	[64]
Lung cancer	Human cancer tissue	Immunosuppression via production of IL-1α	[74]
Hepatocellular cancer	Human blood—healthy donor	Regulation of IL-10 production by CD4^+^ FOXP3- Tregs via ICOS-L upregulation, when exposed to liver tumor lysate	[63]
Gastric cancer	Human cancer tissue	Correlation of pDCs and ICOS^+^ Tregs in peritumoral tissue	[67]
Glioma	Mouse model	Decreased IFNα production upon CpG stimulation, decreased expression of TLR9	[65]
